# Prevalence and sociodemographic correlates of DSM-5 eating disorders in the Australian population

**DOI:** 10.1186/s40337-015-0056-0

**Published:** 2015-04-25

**Authors:** Phillipa Hay, Federico Girosi, Jonathan Mond

**Affiliations:** Centre for Health Research, School of Medicine, University of Western Sydney, Sydney, New South Wales Australia; School of Medicine, James Cook University, Townsville, Queensland Australia; Department of Psychology, Macquarie University, Sydney, Australia, and School of Medicine, University of Western Sydney, Sydney, New South Wales Australia

**Keywords:** Binge eating disorder, Anorexia nervosa, Bulimia nervosa, Population

## Abstract

**Background:**

New DSM-5 diagnostic criteria for eating disorders were published in 2013. Adolescent cohort studies in the Australian community indicate that the point prevalence of DSM-5 eating disorders may be as high as 15% in females and 3% in males. The goal of the current study was to determine the 3-month prevalence of DSM-5 disorders in a representative sample of Australian older adolescents and adults. A secondary aim was to explore the demographic correlates of these disorders, specifically, age, gender, income, and educational attainment and presence of obesity.

**Methods:**

We conducted and merged sequential cross-sectional population survey data of adults (aged over 15 years) collected in 2008 and in 2009 (*n* = 6041). Demographic information and the occurrence of regular (at least weekly over the past 3 months) objective and subjective binge eating, extreme dietary restriction, purging behaviors, and overvaluation of weight and/or shape, were assessed.

**Results:**

The 3-month prevalence of anorexia nervosa and bulimia nervosa were both under 1% whereas the prevalence of binge eating disorder (BED) and sub-threshold BED were 5.6-6.9%. The prevalence of BED including overvaluation of weight/shape was 3%. Other specified and unspecified eating disorders including purging disorder were less common, under 1% to 1.4%. While people with eating disorders were generally younger than others, the mean age was in the fourth decade for anorexia nervosa and bulimia nervosa and in the fourth or fifth decade for all other disorders. Most people with eating disorders had similar household incomes and educational attainments to the general population. People with bulimia nervosa, BED and sub-threshold bulimia nervosa were more likely to be obese than people without an eating disorder.

**Conclusions:**

The findings support the expanded demographic distribution of eating disorders. There is a relatively high prevalence of BED compared to anorexia nervosa and bulimia nervosa. As it is in BED**,** obesity is a very common co-morbidity in bulimia nervosa.

## Background

### Prevalence and sociodemographic correlates of DSM-5 eating disorders in the Australian population

The Diagnostic and Statistical Manual of Mental Disorders criteria for eating disorders were revised in 2013 [1,2]. In the DSM-IV [1] three eating disorders, anorexia nervosa, bulimia nervosa and binge eating disorder (BED- classified under Eating Disorder not Otherwise Specified (EDNOS)), had specific criteria. People who did not meet criteria but nevertheless had an eating disorder were also classified under EDNOS. EDNOS was, however, the most common of all the syndromes in both the clinic and community [3]. A key aim of the revision was to broaden criteria for bulimia nervosa and anorexia nervosa and include BED as a third, formal diagnosis. For bulimia nervosa and BED the specific change was to reduce frequency of binge eating (and for bulimia nervosa compensatory weight-control behaviours) from twice to once weekly and for BED the duration of symptoms was aligned with bulimia nervosa to be 3-months rather than 6-months [4]. EDNOS has also been revised into two new categories: Other Specified Eating or Feeding Disorder (OSFED) and Unspecified Feeding or Eating Disorder (UFED). OSFED has two groups characterized by recurrent binge eating, namely, sub-threshold bulimia nervosa and sub-threshold BED where binge eating frequency and/or the duration of compensatory behaviours are less than weekly for three months. A further overeating subtype of OSFED, night eating syndrome, does not specify that the excessive food consumption entails binge eating episodes.

The DSM-5 revisions have empirical support and are likely to also be introduced in upcoming revisions of the alternate international diagnostic scheme, namely, the International Classification of Diseases [5]. The ICD revisions may however go further in removing the requirement that binge eating episodes entail consumption of an unusually large amount of food, such that people who experience a loss of control over eating but who binge on normal sized food portions – i.e., people who have subjective binge eating episodes – may be eligible for the diagnoses of bulimia nervosa and BED. This is supported by evidence that that the size of the binge is of less clinical utility, diagnostic validity and concern to people who binge than is the experience of being out of control when eating (e.g. Latner and colleagues [6] and Mond [7]).

Criteria for new disorders in DSM-5 depart from those for anorexia nervosa and bulimia nervosa in not requiring the overvaluation of weight/shape or other body image disturbance. However, overvaluation of weight/shape has been argued to have clinical utility as a diagnostic specifier, or perhaps, diagnostic criterion, of BED [8,9]. New disorders such as BED also appear to have different demographic correlates to anorexia nervosa and possibly bulimia nervosa, occurring in older people with a more even sex distribution [10]. There is also potential for people to meet criteria for more than one disorder depending on interpretation of the term “recurrent” in regards to purging episodes. For example, a person who has weekly objective binge eating episodes, less than weekly purging episodes, and overvaluation of shape or weight, may be diagnosed as binge eating disorder (if the less-than-weekly purging is deemed “not-recurrent”) or bulimia nervosa of sub-threshold frequency and/or duration, i.e. OSFED.

There have been a small number of epidemiologic studies of DSM-5 anorexia nervosa, bulimia nervosa, BED, and other disorders involving recurrent binge eating. The community-based 2005 South Australian Health Omnibus Survey (SAHOS) of people aged 15 or more years [11] found a 3-month prevalence of around 1% for bulimia nervosa (84% females), 2% for BED (67% females), 2% for other EDNOS cases (69% females) and 0.3% anorexia nervosa (80% female) using the DSM-5 criteria of weekly frequency of binge eating and extreme weight control behaviours and broader criteria for anorexia nervosa (DSM-5 criteria A and C). These findings were in accord, generally, with international studies which have reported general population point or 12-month prevalence figures for DSM-IV bulimia nervosa of around 2% in women and 0.5% in men and for BED of around 3.5% in women and 2.0% in men [12-15]. Use of different diagnostic schemes, population samples, and assessment instruments likely account for variations in prevalence [16].

Estimates of the lifetime prevalence of DSM-5 eating disorders have since been derived in population-based cohort samples of young female twins by Wade and colleagues [17]. These found a lifetime prevalence of 1-2% for bulimia nervosa and an additional 2% of women met criteria for BED. In a community cohort of 699 adolescent female twins 5.4% of participants had DSM-anorexia nervosa, bulimia nervosa, or BED, 5% OSFED and 4.7% UFED [18,19]. Allen and colleagues [20] reported one-month prevalence of DSM-5 eating disorders in 1383 children of a cohort of 2804 Australian mothers. At age 20, the point prevalence of DSM-5 eating disorders was 15.2% in females (most bulimia nervosa or BED) and 2.9% in males (mostly OSFED). A community-based study of 1584 adolescents in Holland [21] found that 5.7% of the female (95% CI 4.2–7.5) and 1.2% of the male adolescents (95% CI 0.6–2.3) met DSM-5 criteria for an eating disorder in their lifetime, BED being the most common diagnosis followed by anorexia nervosa. Much higher one-month prevalence of OSFED/UFED (43%) and BED (9.9%) were reported in a sample of college age students in the US with rates for anorexia nervosa of 0.1% and bulimia nervosa 1.2% [22]. Large international variation in prevalence estimates support the need for further, population-based research.

In addition to informing community prevalence, epidemiologic surveys can inform the socio-demographic distribution of eating disorders free from the selection bias inherent in clinic samples, for example, access and availability of care [10]. Sex, age and socio-economic status are particular demographic correlates whose rates have been thought to vary across eating disorder diagnostic groups [10] with aetiologic and health care provision import. For example the sex bias towards women has been thought to be relevant to greater social pressures on women to be thin, exemplified in the book “Fat is a feminist issue” [23]. Others have relevance to access to care. For example, limited financial capacity to pay for health care and in men embarrassment with having a perceived “female” problem are important barriers to seeking help [24,25] and the misperceptions that eating disorders are uncommon in lower socio economic groups or men may thereby contribute to deficits in health care provision to these groups.

The goal of the current study was to determine the 3-month prevalence of DSM-5 disorders in a representative sample of Australian older adolescents and adults. Secondary aims were to compare the prevalence of bulimia nervosa and BED according to DSM-IV and DSM-5 criteria, and of BED with and without overvaluation as a diagnostic criterion, and to delineate the demographic correlates of eating disorder diagnoses, specifically, age, gender, household income and education level distributions.

## Methods

### Study design and participant recruitment

Data in this study were collected from questions that were embedded in two independent cross-sectional Health Omnibus Surveys, conducted in 2008 and in 2009. The Health Omnibus Survey is conducted annually by Harrison Health Research [26], under the auspices of the South Australian Health Commission, and involves face-to-face interviews of a representative sample of the South Australian population. The interviews are respondent-based and ask a range of both demographic and health-related questions.

The sample selection and interview procedures were similar in both survey years. In both years, metropolitan and rural “collector” districts in South Australia were identified based on a probability proportional to size sampling procedure, according to the Australian Bureau of Statistics 2006 Census data. Within each district, 10 dwellings were chosen to conduct interviews in. The person to be interviewed within each dwelling was the person who was older than 15 years and had their birthday most recently. The samples were non-replacement, and up to six visits were made to conduct an interview with the designated participant in each designated dwelling. Interviews were conducted from February until July in 2008 and from September until December in 2009. In total 6041 people (range 15–96 years) were interviewed. To ensure feasibility and participant understanding of questions over 50 interviews were conducted in a pilot period during January 2008 and August 2009.

In 2008 and 5000 and in 2009 5200 households were selected and 3034 and 3007 interviews were conducted respectively. The response rates were 62.8% in 2008 and 59.3% in 2009 with the majority reason for non-participation was refusal (n = 1110 in 2008 and 1376 in 2009).

### Ethics statement

Participants provided verbal rather than written informed consent, due to the practicalities of carrying out a large-scale survey and the low risk nature of the survey content. The 2008 survey was approved by the Research Ethics Committee of the Government of South Australia, Department of Health, and the 2009 survey was approved by the University of Adelaide HREC.

### Assessment of eating disorder features

Four eating disorder features were assessed, namely: binge eating (objective and subjective); purging; extreme dietary restriction; and the overvaluation of weight or shape. The questions in the surveys that were to elicit information regarding the presence of these features from participants were based on diagnostic questions from The Eating Disorder Examination [27]. Objective binge eating was assessed in both surveys by asking participants whether they regularly felt that they ate ‘*an unusually large amount of food*’ and at the same time experienced a feeling of being ‘*out of control*’. Subjective binge eating was measured similarly to objective binge eating except that the amount of food consumed was defined as not unusually large. Purging was assessed in both surveys by asking participants whether they regularly used laxatives, diuretics (water tablets), or self-induced vomiting as a means to control their weight or shape. Extreme dieting was assessed in both surveys by asking participants whether they have regularly gone on a ‘*very strict diet’* or ‘*hardly eaten anything at all’* in order to influence their weight or shape. The term ‘regular’ used in each of these questions was defined as the behavior having occurred at least once per week over the three months prior to the interview. Participants were asked the level of importance they placed on weight and/or shape in determining their self-evaluation on a 6-point scale. Answers to this question were grouped into two categories, namely, overvaluation absent (participants who reported none to moderate importance of weight and shape in determining self-evaluation) and overvaluation present (participants who reported extreme to supreme importance of weight and shape in determining self-evaluation, at a level of 4, 5 or 6). Current body mass index (BMI, kg/m^2^) was calculated based on self-reported weight and height.

For the purpose of creating mutually exclusive diagnostic categories, the following criteria were used to define the DSM-5 diagnostic terms: anorexia nervosa was defined as participants with BMI < 18 and weight/shape overvaluation at a level of 4, 5 or 6 (i.e. DSM-5 criteria A and C); bulimia nervosa was defined as participants with weekly objectively large binge eating episodes and purging episodes or strict dieting occurring weekly and weight/shape overvaluation at a level of 4, 5 or 6 and BMI 18 or more (i.e., all the DSM-5 criteria); BED was defined as participants with weekly objectively large being eating episodes, no weekly purging and BMI 18 or more (i.e., DSM-5 criteria A, D, E); OSFED bulimia nervosa-type (bulimia nervosa of sub-threshold frequency and/or duration) was defined as less than weekly objective bulimic episodes, weekly purging or dieting weekly and weight/shape overvaluation at a level of 4, 5 or 6; OSFED BED-type (BED of sub-threshold frequency and/or duration) was defined as less than weekly objective bulimic episodes, no weekly purging or dieting, and not meeting criteria for AN; OSFED ‘purging disorder’ was defined as having weekly purging episodes, no objectively large binge eating episodes and not meeting criteria for anorexia nervosa; and UFED was defined as having weekly subjective and/or objective binge eating episodes, or weekly purging episodes, or strict dieting with overvaluation, or strict dieting with BMI <18, and not meeting criteria for another diagnostic category.

### Data analysis

Data from the two surveys, which comprised distinct samples interviewed 12 months apart, were merged for the purpose of the current study. Data in both years were weighted by the inverse of the individual's probability of selection, then re-weighted to benchmarks derived from the Estimated Resident Populations at 30th June 1994, by age, sex and Local Government Area, from the Australian Bureau of Statistics (Catalogue No 3204.4). Weighted data are reported in this paper. Prevalence data are reported as percentages with 95% C.I. calculated using the Newcombe-Wilson [28] method without continuity correction using an Excel syntax. Statistical differences between groups were tested using oneway ANOVA with Tukey post-hoc for parametric data and Kruskal-Wallis (K-W), Mann–Whitney U (M-W) and Chi-sqare (χ^2^) tests as appropriate for ordinal or categorical data. All statistical analysis was conducted using SPSS version 22.

## Results

### Identification of cases

The 3-month point prevalence of any form of a DSM-5 eating disorder or disordered eating was 16.3% (N = 985 95% CI 15.4 to 17.3). The prevalence of each disorder is shown in Table 1. Together anorexia nervosa (n = 28), bulimia nervosa (n = 40) and BED (n = 337) accounted for 6.7% of community cases (95% CI 6.1 to 7.4). As can be seen, the prevalence of bulimia nervosa and BED was reduced by nearly 50% if the DSM-IV twice weekly frequency of behaviours is applied and the prevalence of BED and sub-threshold BED was reduced by a third if overvaluation as a diagnostic specifier is applied.

**Table 1:** **Point (3-month) prevalence of DSM-5 eating disorders (n = 6041)**

	**n**	**%**	**95% C.I.**
Anorexia Nervosa	28	0.46	0.32;0.67
Bulimia Nervosa	40	0.66	0.49;0.90
Bulimia Nervosa with DSM-IV frequency criteria^1^	20	0.33	0.21;0.51
Binge Eating Disorder (BED)	337	5.58	5.03;6.19
BED with weight/shape overvaluation	180	2.98	2.58;3.44
BED with DSM-IV frequency criteria^1^	146	2.42	2.06;2.84
Sub-threshold Bulimia Nervosa	42	0.70	0.51;0.94
BED Sub-threshold	418	6.92	6.31;7.59
BED Subthreshold with weight/shape overvaluation	58	2.62	2.24;3.05
Purging Disorder	35	0.58	0.42; 0.80
Unspecified Feeding or Eating Disorder	85	1.41	1.14;1.74

^1^DSM-IV frequency criteria were twice weekly for both binge eating behaviours.

### Demographic analyses

The demographic correlates of the disorders are shown in Table 2. There was an overall age difference between groups (one-way ANOVA F = 26.32, df = 7, p < 0.001; Tukey post-hoc p < 0.05). People with anorexia nervosa, bulimia nervosa, BED, sub-threshold bulimia nervosa, sub-threshold BED and UFED were significantly younger than people without an eating disorder. People with purging disorder were significantly older than people with anorexia nervosa, bulimia nervosa, sub-threshold bulimia nervosa and sub-threshold BED.

**Table 2:** **Sociodemographic characteristics of participants with DSM-5 eating disorders (total n = 6041)**

	**Age/years**	**Gender**	**Annual household**	**Highest education**	**Obese (BMI ≥ 30)**
	**Mean (SD)**	**n (%) female**	**Median income level**	**Median level**	**n (%)**
N gave information	6041	6039	4761	6032	6039
Anorexia nervosa	32.8 (18.7)	24 (83%)	$60-80 K	Post high school	n.a.
Bulimia nervosa	34.5 (12.9)	27 (69%)	$40-50 K	Trade/Certificate	16 (41%)
Binge eating disorder	39.7 (16.9)	191 (57%)	$50-60 K	Post high school	126 (37%)
OSFED					
Sub-threshold BN	33.5 (12.5)	31 (74%)	$60-80 K	Trade/Certificate	17 (41%)
Sub-threshold BED	38.8 (16.4)	229 (55%)	$60-80 K	Trade/Certificate	89 (21%)
Purging Disorder	49.0 (15.9)	27 (77%)	$50-60 K	Trade/Certificate	10 (29%)
UFED	40.0 (15.5)	61 (73%)	$80-100 K	Trade/Certificate	11 (13%)
No eating disorder	47.0 (19.0)	2489 (49%)	$50-60 K	Post high school	854 (16.8%)
All	45.6 (18.9)	3079 (51%)	$60-80 K	Post high school	1123 (19%)

Data reported in this table are weighted for South Australian norms; BN = bulimia nervosa, BED = binge eating disorder, OSFED = Other Specified Eating or Feeding Disorder, UFED = Unspecified Feeding or Eating Disorder.

There was an overall difference in sex distribution with a significant female majority in all groups excepting those with sub-threshold BED (Chi square χ^2^ = 64.3, df = 7, p < .001).

Income was grouped into 9 levels from less than $12000 to more than $100000 Australian dollars per year. The median income level for the whole sample was $6000-80000 per year (IQ range $30000-40000 to $80000-100000). All participants with an eating disorder had a higher median income compared to people without an eating disorder (M-W U Z = −2.97, n = 4704, p = .003).

There was an overall difference in median income levels between eating disorder groups and people without an eating disorder (K-W test χ^2^ = 25.8, df = 7, p = .001). Individuals with sub-threshold BED and UFED had higher incomes than people without sub-threshold binge eating or UFED respectively (M-W U Z = −3.76, p < .001, M-W U Z = −2.55, p = .011) and this was whether overvaluation was or was not a diagnostic specifier for sub-threshold BED (M-W Z = −2.81, p = .005 and M-W Z = −2.67, p = .008 respectively). No other eating disorder or disordered eating group differed significantly in median incomes from people without an eating disorder (i.e. all those without the respective eating disorder or disordered eating group).

Education was grouped into five levels of: currently at school, left school with no qualification yet attained, attained a certificate or trade level qualification, and attained a bachelor’s degree or higher. The median level of education in the whole sample was having left school without a qualification. Level of education did not differ across groups (K-W χ^2^ = 6.93, df = 7, n = 5338, p = .44).

There was an overall between group difference in distribution of obesity (χ^2^ = 126.96, df = 7, p < .001, n = 6039) with a lower proportion of obesity found in individuals without an ED and in those with sub-threshold BED, purging disorder and UFED (p < .05). This contrasted with a higher proportion of obesity in individuals with bulimia nervosa, BED and sub-threshold bulimia nervosa.

## Discussion

The present study found a 3-month point prevalence of 16.3% for any DSM-5 eating disorder and this was largely due to the numbers of people with BED (6%) and sub-threshold (or OSFED-type) BED (7%). In contrast anorexia nervosa and bulimia nervosa each occurred in less than 1% of the general population. Rates for BED were substantially lower (3%) if overvaluation was included as a diagnostic criterion, as it was in the 2005 Australian survey [11]. The figures for BED with overvaluation are commensurate with those found in the 2005 Australian survey and for DSM-IV BED in international studies [13]. The high prevalence of BED and OSFED- type BED in the present study is consistent with figures in adolescent and young adult cohort samples [19-21].

As found by others [22] the DSM-5 criteria has reduced numbers of people in the OSFED/UFED categories compared to the former EDNOS DSM-IV [1] category. This can be explained by the broadening of diagnostic criteria for all three main eating disorders, anorexia nervosa, bulimia nervosa and BED. Decreasing the frequency criteria for bulimia nervosa and BED behaviours in particular has resulted in notable increases in the prevalence of these disorders. This should assist health care planning and provision of specific evidence based treatment services for these eating disorders and compares with the previous situation where it was difficult to plan specific service needs of people with EDNOS due to the lack of a specific evidence base for treatments of this heterogenous category [29].

The study found that people with an eating disorder are generally younger than people without an eating but this was most apparent in those with anorexia nervosa or bulimia nervosa, who had mean age in the fourth decade. People with BED, OSFED or UFED had mean ages in their mid-life years and people with purging disorder had a mean age similar to that of the general population. Female predominance was most marked in people with anorexia nervosa and was least pronounced in people with disordered eating characterised by recurrent binge eating where rates approached sex equivalence. Median incomes were higher for people with an eating disorder compared to the general community but on post-hoc analyses only in individuals with sub-threshold BED and UFED. Educational attainment was similar for people with eating disorders compared to that of the wider community. Taken together these findings support the expanded distribution of eating disorders in terms of demographic characteristics, including increasing numbers of males in the community with these disorders, particularly those characterised by recurrent binge eating [10,13].

We also found that obesity was a common feature of individuals with eating disorders. People with bulimia nervosa, BED and sub-threshold bulimia nervosa were more likely to be obese than people who did not have these disorders whilst those with UFED, purging disorder and sub-threshold BED had lower rate of obesity. The last three groups were those also with lower levels by definition of binge eating. Those with bulimia nervosa and BED had by definition the highest levels of binge eating. However, it is unclear why the proportion of people with obesity was higher for those with sub-threshold bulimia nervosa compared to sub-threshold BED (21% versus 41%) as the former by definition in this study (in order to create distinct categories) engaged in regular weight control behaviours which might have been thought to reduce levels of obesity. Alternatively, individuals who are obese more frequently engage in weight control behaviours [30]. The findings support recent calls for a change in the assessment and care of bulimia nervosa, as in BED, to encompass weight management as well as eating disorder management [31].

Limitations of the present study relate primarily to the assessment of eating disorder features which was by structured interview by lay interviewers and not based on clinical interviews. Weight control behaviours of less than weekly frequency were not determined and there was no assessment of excessive exercise among these behaviours. A further limitation of the assessment method was that people with weight/shape overvaluation and both less than weekly binge eating and less than weekly weight control behaviours were not able to be identified and included in the sub-threshold bulimia nervosa group and this deficit may have inflated the rate of obesity in people with subthreshold bulimia nervosa (as weight control behaviours are more common in people who are overweight or obese [30]). In addition the use of a respondent-based interview may have contributed to over-reporting of frequency of binge eating and eating disorders characterised by compulsive exercise, night eating syndrome and ARFID were not assessed. Because distress and the other diagnostic specifiers of binge eating in the DSM-5 criteria for BED and the criterion B for DSM-5 anorexia nervosa were not assessed it is possible the prevalence of both BED and anorexia nervosa was inflated. This is difficult to know because these criteria are rarely used for research purposes. Further, lower numbers in some groups, especially anorexia nervosa, may have contributed to Type II error in secondary analyses of between group differences. Finally, the study is based in Australia and findings may differ in other nations.

## Conclusions

Eating disorders are common in the community where there is a relatively high prevalence of BED compared to anorexia nervosa and bulimia nervosa. The findings support the expanded demographic distribution of eating disorders and the need for services to be inclusive of males as well as females and people of all ages, incomes and education. As it is in BED, obesity is a common co-morbidity in bulimia nervosa and consideration needs to be given to addressing weight disorder as well as the eating disorder in assessment and treatment.
